# De-Extinction

**DOI:** 10.3390/genes9110548

**Published:** 2018-11-13

**Authors:** Ben Jacob Novak

**Affiliations:** 1 Revive & Restore, Sausalito, CA 94965, USA; ben@reviverestore.org; Tel.: +1-415-289-1000; 2 Department of Anatomy and Developmental Biology, Monash University, Clayton, Victoria 3800, Australia; 3 Australian Animal Health Laboratory, Commonwealth Scientific and Industrial Research Organization, Newcomb, Victoria 3220, Australia

**Keywords:** de-extinction, precise hybridization, evolutionarily torpid species, proxy, passenger pigeon, woolly mammoth, heath hen, revive & restore

## Abstract

De-extinction projects for species such as the woolly mammoth and passenger pigeon have greatly stimulated public and scientific interest, producing a large body of literature and much debate. To date, there has been little consistency in descriptions of de-extinction technologies and purposes. In 2016, a special committee of the International Union for the Conservation of Nature (IUCN) published a set of guidelines for de-extinction practice, establishing the first detailed description of de-extinction; yet incoherencies in published literature persist. There are even several problems with the IUCN definition. Here I present a comprehensive definition of de-extinction practice and rationale that expounds and reconciles the biological and ecological inconsistencies in the IUCN definition. This new definition brings together the practices of reintroduction and ecological replacement with de-extinction efforts that employ breeding strategies to recover unique extinct phenotypes into a single “de-extinction” discipline. An accurate understanding of de-extinction and biotechnology segregates the restoration of certain species into a new classification of endangerment, removing them from the purview of de-extinction and into the arena of species’ recovery. I term these species as “evolutionarily torpid species”; a term to apply to species falsely considered extinct, which in fact persist in the form of cryopreserved tissues and cultured cells. For the first time in published literature, all currently active de-extinction breeding programs are reviewed and their progress presented. Lastly, I review and scrutinize various topics pertaining to de-extinction in light of the growing body of peer-reviewed literature published since de-extinction breeding programs gained public attention in 2013.

## 1. Introduction

The term de-extinction first gained significant public interest in March 2013 thanks to a series of live-streamed talks at the TEDxDeExtinction event, organized by the conservation non-profit organization Revive & Restore and hosted by National Geographic Society [1], which simultaneously published an accompanying de-extinction cover story in the March issue of National Geographic magazine. It was at this event that Revive & Restore announced its flagship de-extinction project, “The Great Passenger Pigeon Comeback” [2]. De-extinction was introduced as a means to “undo” historic extinctions by restoring new versions of extinct species to their former habitats. The idea continues to spur ethical, social and technological debates in the media, among scientists at public events and even at special symposia at academic conferences. The ensuing popularity of de-extinction has resulted in hundreds of press articles, online videos and several television documentaries. To date, eleven popular books have been published with chapters on de-extinction [3,4,5,6], or entirely on de-extinction [7,8,9,10,11], including a biopic novel on George Church’s work on woolly mammoth de-extinction [12] and one, very creative, fictional take on passenger pigeon de-extinction self-published by Ryan Patrick Lewis, 11 years old at the time of writing [13]. In academia, de-extinction has been featured in several special-topic textbooks [14,15,16]. In peer-reviewed literature, de-extinction has been the subject of several special journal issues and many independent articles, totalling 66 published papers. The unifying trends in all of this literature are confusion over what de-extinction means, the processes by which it can be achieved and its intended purposes.

Incoherencies in de-extinction literature can be attributed to the uncomfortable philosophical and scientific reality the topic raises: what exactly is a species? And, even more complicated a question, what is extinction? The conservation community prefers to have these concepts well defined and well understood but in truth, they have always been hugely complex biological and philosophical concepts [17,18]. The accepted definitions of species and extinction are key factors shaping conservation paradigms. For wildlife regulation, both the concept of a species and of extinction must be well defined. Any acknowledgement that both of these concepts are fluid upsets existing conservation paradigms, which is the very disruption that de-extinction interjects. However, it is well understood in the scientific community that both of these concepts have gradations: species, subspecies, distinct populations, ecotypes; functionally extinct, locally extinct, extinct in the wild, globally extinct. The challenging fluidity of these concepts becomes clear when one considers that in some cases species can go extinct by evolving into a new species (termed anagenetic speciation [18]); as species A evolves to species B, species A ceases to exist. However, the lineage is unbroken, so is species A actually extinct? Is species B actually a distinct and new species? Hybridization and its role in evolution and extinction, is another complicating factor. If species C hybridizes with species D, resulting in the extinction of all individuals exhibiting species C’s phenotypes but leaves its genotypes reproducing in individuals exhibiting species D’s phenotypes, is species C actually extinct? Is species D now a new species despite the fact it is visibly unchanged? How much change is required for one species to be considered a different species from its historically-recognized form? What if the genetics of a species remain unchanged but its microbiome and behaviour change; is it now a newly evolved species B? If so, does that mean species A is now extinct despite no changes to its genetic lineage? These are all questions conservation passes over but that de-extinction brings to the forefront.

Nowhere are these questions more problematic than when considering the current working definition of de-extinction, which a special committee of the International Union for the Conservation of Nature delineated in a 2016 document outlining the guiding principles for de-extinction considerations and practice [19]. The International Union for the Conservation of Nature (IUCN) guidelines defined de-extinction as the generation of proxies of extinct species that are functionally equivalent to the original extinct species but are not “faithful replicas.” Proxy is meant to address the fact that the original extinct species’ lineage can never be fully recovered (explained in more detail in Section 5) and therefore only an altered version of a living species can substitute (proxy) for the extinct species.

The IUCN guidelines outlines three methods to generate proxies of extinct species, which has been echoed by many independent authors: selective breeding, genome editing and cloning. By including cloning, authors have contended that the birth of a cloned Bucardo in 2003 was the first de-extinction [19,20,21,22,23], however short-lived as the cloned Bucardo died seven minutes after birth due to a lung-deformity [24]. This is not the first de-extinction, nor is a cloned Bucardo by any means a “proxy.” It is, in fact, a “faithful replica,” meaning that the IUCN committee authors defied the very definition they set. Considering a cloned organism, a proxy has been built on the argument that a cloned member of an extinct species is epigenetically different from its historic ancestors—an argument made solely in the vacuum of de-extinction discourse which, if we accept this argument, casts an alarming realization over all of conservation. If a cloned Bucardo is a proxy of a Bucardo, then every species epigenetically altered by human activities is now extinct and has been replaced with anthropogenic proxies. This means every recovery facilitated by translocation, captive breeding, habitat restoration and so forth, are not recoveries at all but have rendered species extinct and substituted them with new forms. We can acknowledge this reality, or we can consider that our definitions of de-extinction and extinction are flawed and work through the new realizations that de-extinction practice presents.

In this paper I present a comprehensive biological and ecological definition of de-extinction that solves the issue inherent in the IUCN definition’s inclusion of cloning as a de-extinction technique, thereby elucidating a new classification of endangerment for special cases of species recovery (outlined in Section 4). Expounding upon this clarified view of de-extinction, I discuss the technical limitations of de-extinction in brief and offer a scheme for classifying proxy populations as well as prioritizing de-extinction candidates. I also review the world’s active de-extinction breeding programs (Table 1), some of the leading criticisms of de-extinction in light of the information presented and critique the current body of literature regarding de-extinction in the hopes of establishing a coherent de-extinction dialogue.

## 2. Defining De-Extinction: Replacement by Proxy versus Assisted Recovery

A corrected definition of the mode of de-extinction outlined by the IUCN is this: de-extinction is the ecological replacement of an extinct species by means of purposefully adapting a living organism to serve the ecological function of the extinct species by altering phenotypes through means of various breeding techniques, including artificial selection, back-breeding and precise hybridization facilitated by genome editing. The goal of de-extinction is to restore vital ecological functions that sustain dynamic processes producing resilient ecosystems and increasing biodiversity and bioabundance.

**Figure 1:**
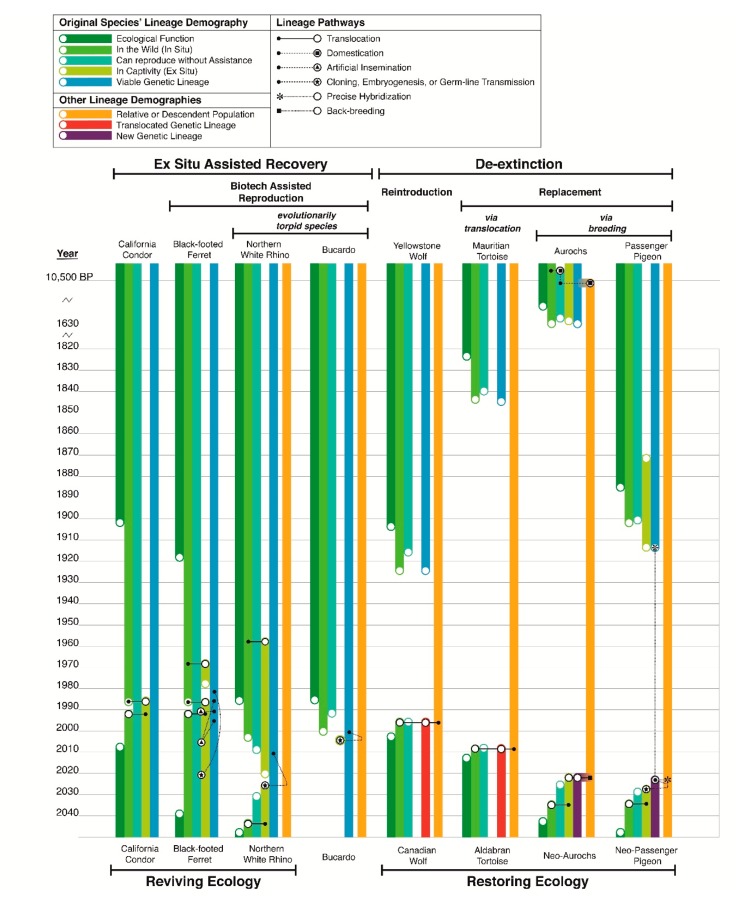
Graphic representation of ecological and biological lineages of species through time to compare and contrast examples of assisted recovery and de-extinction. The examples display the parallel nature of recovery and de-extinction techniques with the restoration of ecological function ensuing from the return or introduction of species to the wild (in situ). As can be seen, the genetic lineage of recovered species never breaks but persists, while in de-extinction efforts the original historic species, subspecies, or population is extinct or altered, requiring a new form to replace it. For the aurochs, it is clear that the lineage is unbroken but has been altered through historic and modern day breeding. For the passenger pigeon, some of the genetics from the historic lineage are recovered in the hybrid proxy lineage. The black-footed ferret is an excellent example of an extant species through which biotechnology has been used to assist recovery; cryopreserved semen has been used to retrieve historic genetics and maintain diversity in the population [29]. Revive & Restore has initiated work to test cloning in black-footed ferrets from cryopreserved cells at the SDZGFZ, which may recover the genetics of two deceased individuals that would represent new founders to the population [30]. Progress for each program is predicted beyond the present to show the intended goals of nascent projects. In the case of northern white rhinoceros recovery, the projected timeline shows a break in the ex situ lineage representing the eventual death of the last two surviving adults, which may or may not occur before a new generation of offspring are produced via stem cell embryogenesis or cloning.

This form of de-extinction in practice is simply a continuation of restoration ecology that began in the 1830’s with the restoration of the locally extinct capercaillie grouse throughout Scotland by means of translocating individuals from extant populations elsewhere [25]. Restoring locally extinct populations through means of translocation has become an increasingly implemented conservation intervention in recent decades [26], the most famous of which being the return of wolves to Yellowstone National Park after a seventy year absence [27]. From a population genetics perspective, this reintroduction can be considered the introduction of a proxy population, as present day Yellowstone wolves are not descended from the locally co-evolved population that went extinct. They are individuals from other lineages; in this case a separate subspecies (Figure 1). The replacement of an extinct species with another species entirely has also been performed to achieve ecological restoration in recent decades, a well-known case being the introduction of Aldabran giant tortoises to satellite islands of Mauritius to serve the ecological function of the extinct Mauritian giant tortoise [26]. In this context, replacement by proxy is a conservation discipline nearly two centuries old that is a maturing disciplinary practice both scientifically and socially [28]. If we consider that the primary definition and purpose of de-extinction is simply the replacement of an extinct population with an extant functionally equivalent population (regardless of the extant population’s source), then all forms of reintroduction and replacement can be termed a type of de-extinction. Furthermore, the only innovation modern de-extinction makes on traditional ecological restoration practice is that advanced breeding techniques are used to create the replacement population (Figure 1).

Under this encompassing definition, the first completely successful de-extinction effort via breeding is the restoration of the North American eastern peregrine falcon, *Falco peregrinus anatum*, population following its localized extinction in the 1960’s. It was and is, debated that the locally extinct population of *F. p. anatum* was actually distinct from surviving populations to the west and north [31]. For this reason it was argued by some that extant *F. p. anatum* falcons may not successfully colonize the eastern United States given their lack of adaptation to the local environment. The recovery program therefore conducted a blind eco-evolutionary experiment to account for such knowledge gaps, releasing individuals interbred from different subspecies [32] to establish a genetically mixed founding stock and allow the environment to select the most successful genotypes/phenotypes. Today, because of the intentional release of hybridized individuals, peregrine falcons have successfully recolonized many urban, suburban and some wilderness areas throughout the eastern United States. This new anthropogenically-produced population is genetically different from all extant and extinct subspecies due to its breeding. Therefore, under the definition presented in this paper, the restoration of peregrine falcons to the eastern United States was a modern de-extinction facilitated by breeding.

The reintroduction of a species that has gone extinct in the wild (completely throughout its range)—such as the California condor, the whooping crane, the scimitar-horned oryx, the black-footed ferret, or the kihansi spray toad—is not de-extinction, nor have any authors argued that such efforts represent de-extinction. Despite the fact that all these species went extinct in their habitats and are inarguably altered epigenetically, their return to the wild is not a form of de-extinction because the individuals reintroduced are not genetically or intentionally phenotypically altered in any way: their lineage is unbroken and unaltered, as shown by the examples of the black-footed ferret and California condor in Figure 1. This is considered assisted recovery. It is separate from reintroduction via translocation, as the individuals reintroduced are from captivity rather than captured from wild populations to be relocated. From an ecological perspective, assisted recovery and de-extinction eventually achieve the same goal: the restoration of ecological function after a period of absence (dark green bars in Figure 1), however, often assisted recovery is motivated to save a species as an evolutionary entity as well as an ecological entity, while reintroduction via translocation only restores ecological entities. The key difference is that in assisted recovery the original population’s genetic lineage is maintained. It is this fact that precludes the cloning of the Bucardo as de-extinction and moves this effort securely under the umbrella of assisted recovery (Figure 1). The caveat with discerning de-extinction via reintroduction versus recovery via reintroduction is a matter of the taxonomic level in question—species, subspecies, or population. In the case of populations, most populations of a species go extinct and cannot be recovered during a population bottleneck, meaning that a single surviving population then replaces the extinct populations after reintroduction; for example, all reintroduced Black-footed ferret populations are derived from one remnant population, therefore it is arguable that most living populations have been replaced by another population. However, at the species level, black-footed ferrets never went extinct and their reintroduction to the wild is a true recovery, which is generally the accepted case among conservationists. However the opposite is regarded for the Yellowstone Wolf population, which is seen as a replacement of an extinct population, presented as “translocation for rewilding” by Seddon et al. [26] but at the species level wolves were never extinct and this effort could be argued to be a case of recovery, or “translocation for conservation” according to Seddon. The lines between assisted recovery and de-extinction blur for species with highly variable, distinct populations such as the wolf but are more concrete for species with less variability, such as the black-footed ferret.

If a species can go extinct in the wild and then be reintroduced from a captive population, why cannot a fully extinct species return to its habitat? As previously stated, it has been argued that because clones of extinct species will inherently be epigenetically and behaviourally different from their ancestors, they are proxies and not the original species. Under this arbitration, California condors, black-footed ferrets and others are all proxies of now extinct “authentic and natural” versions of their kind. By asserting that human alteration to a species fundamentally changes its classification ignores the fact that species have co-evolved with humans for thousands to hundreds of thousands of years globally. There is no species alive today that has not adapted in some way to human activities of the past and thus become changed from its pre-human contact state. If conservation is to view such reintroduced species as the California condor and the black-footed ferret as proxies, the ex situ and also in situ recoveries of hundreds of species will be incorrectly and unnecessarily reclassified, when in biological reality the original lineage remains the same.

There are scientists that argue that a clone of an extinct species is not a “faithful replica” because the cloned individual harbours the mitochondrial DNA of a related species. This is an equally shaky argument, as it would force us to reclassify dozens of hybrid populations—for example, under this consideration, all of the brown bears of the Admiralty, Baranof and Chichagof islands cannot be considered brown bears because they ubiquitously possess polar bear mitochondria [33]. These bears are not only phenotypically brown bears, with no morphological phenotypes in common with polar bears but are also overwhelmingly genetically brown bear—with less than 1% of their nuclear DNA originating from polar bear ancestry. Hybridization of this sort is being discovered widely among populations of wild species. These mismatched mitochondria are hardly cause for reclassification, so why should mito-nuclear mismatch matter in terms of defining a species’ recovery, even recovery from extinction?

## 3. Biotechnology Changes the Concept of Extinction

The issue of redefining extinction in the era of biotechnologically-assisted reproduction is one of ontogeny. Every organism on the planet goes through a single-cell ontogenetic stage, whether asexually or sexually reproducing. However brief, this stage is necessary to complete an organism’s life cycle. Any single cell that can be coaxed into progressing to later ontogenetic stages is a potential individual for a species. For sexually reproducing species, this stage is the single-celled zygote. Thanks to biotechnology, zygotes of various mammalian species have been transferred from their biological mother to a surrogate pregnancy recipient or produced via in vitro fertilization and then implanted to a surrogate mother. Such zygotes have been cryopreserved and implanted months to years later, producing viable offspring. This establishes the grounds that cryopreserved zygotes—single-celled ontogenic stages—are potential individuals for a species. If every multi-celled ontogenic stage (juveniles to adults) of a species were to die off, leaving the species extinct by conventional definition, it is not implausible to assume that cryopreserved zygotes could recover the species if suitable surrogate mothers existed to gestate and bring those zygotes to term. When considering new biotechnologies, such as cloning and even the production of embryos from induced pluripotent stem cells (stem cell embryogenesis), then a number of cell types, not just zygotes, are reproductively competent. From this perspective, the Bucardo population consists of zero multi-celled individuals and several million single-celled clonal individuals currently cryopreserved.

This ontogenetic population assessment is not only pertinent to species currently considered extinct. The Northern White Rhinoceros is currently incapable of reproducing without biotechnological assistance but is not considered globally extinct. It is considered functionally extinct. The Northern White Rhinoceros population is comprised of two multi-celled adult individuals still living but also millions of single-celled individuals of a dozen genetic lineages [34]. An ambitious program is using these cryopreserved cells to recover the species via stem-cell embryogenesis [35,36], with cloning as a secondary recovery pathway. It is likely that an entirely new generation of Northern White Rhinoceros will be born in the coming decade, possibly after the last surviving adults die (hypothetical outcome shown in Figure 1). Is the species considered completely extinct in the interim between the death of the last adult and the birth of the first new calf? The newborn generation will be from the same genetic lineage, meaning this will not be de-extinction. Is it resurrection? The Bucardo, currently considered extinct with no living individuals, cannot reproduce without biotechnological assistance either. In light of biotechnology, is the Bucardo extinct or simply functionally extinct like the Northern White Rhinoceros? The cloning of a Bucardo is proof that individuals could be recovered with biotechnologically-assisted reproduction, which under Siipi and Finkelman’s consideration of extinction would mean that the Bucardo is not extinct, or “non-extinct” in their terminology [17]. Are the Bucardo and the Northern White Rhinoceros different variations or degrees of extinction and endangerment, or similar cases? While there is no doubt a myriad of philosophical points to argue either view, biologically speaking they are identical cases of reproductive states. The unification of the extinct Bucardo and functionally extinct Northern White Rhinoceros under the same reproductive scenario demands the delineation of a new class of endangerment, removing these species from the categories of extinction.

## 4. Evolutionarily Torpid Species

I propose that reproductively competent single cells be considered ontogenetic individuals of a species and offer a new classification for particular cases when the multi-celled ontogenetic individuals of a species can no longer reproduce without assistance. These species are “evolutionarily torpid.” They are not extinct because they persist as single-celled individuals but they are not actively reproducing, meaning they are not evolving. Species adapt to their environments, whether in situ or ex situ, by undergoing selection when reproducing. A species that ceases to reproduce ceases to evolve, entering an unchanging dormant state (torpor). Only a handful of evolutionarily torpid species exist (Table 2) and the cloning of the Bucardo represents the first time an evolutionarily torpid species has been recovered. The Bucardo was cloned from cultured fibroblasts that had been cryopreserved for less than three years at the time. The generation of multi-celled embryos of the gastric-brooding frog represents a recent partial success in recovering an evolutionarily torpid species, a feat made more impressive by the fact that embryos were generated from uncultured cells from non-cryopreserved frozen tissues over thirty years old [37]. Currently, the Northern White Rhinoceros stands to be the first probable recovery of a species from an evolutionarily torpid state.

Although currently rare, species slipping into evolutionarily torpid states could become more frequent in the future as populations of many endangered species continue to decline while cryopreservation collections at zoos and museums continue to grow. The San Diego Zoo Global’s Frozen Zoo^®^ has cultured cell lines of over 1000 species [39]. If breeding populations of any of these species were to die off globally, these preserved cell lines would offer hope of recovery—rendering those species evolutionarily torpid rather than completely extinct. The gradations of extinction can now be termed as locally extinct, extinct in the wild (when individuals survive in captivity), functionally extinct (when reproduction has ceased but reproductively viable resources do not exist), evolutionarily torpid (when reproduction has ceased but reproductive resources persist) and globally extinct (when no reproductive material of any kind exists and the last living individual has died). Such classifications of extinction are more helpful for conservation practice and intervention than a binary concept of extinction and survival.

## 5. Limitations of De-Extinction via Breeding

A truly extinct species, subspecies, or genetically unique population, for which no viable cells are preserved cannot currently and may never, be resurrected due to technological and biological limitations. When it comes to the various techniques of de-extinction breeding, most are incapable of producing proxies of the vast majority of extinct species. Back-breeding is only applicable to extinct species for which there are living descendants. Artificial selection is likewise limited to cases in which variation in an extant species’ phenotypes can be selected to reproduce phenotypes parallel to extinct species. Only precise hybridization, via genome editing, is applicable to the majority of de-extinction candidates. The requirements for precise hybridization are the genome sequence of the extinct species and an extant template species whose genome can be edited to express alleles of the extinct species, producing a hybrid proxy that expresses the phenotypes of the extinct species derived from those edited alleles. Paleogenomicists have sequenced genomes of fossils dating as far back as 700,000 years BP (before present) [42], making most species of the late Pleistocene and the Holocene potential candidates for precise hybridization. In general, the preserved skins and bones archived throughout the world’s museums are resources for sequencing genomes of recently extinct species. A number of extinct species have already had their genomes fully or partially sequenced (Table 3).

De-extinction is not only limited by the half-life of DNA [61] but also the technological ability to synthesize it. The longest synthesized DNA sequence to date is 770 kb [62]. Eukaryotic chromosomes can be hundreds of millions of base pairs long, making whole genome synthesis infeasible at present, which is why de-extinction practitioners are employing a strategy of selective gene editing to accomplish their goals. For example, while the genome of the woolly mammoth, *Mammuthus primigenius*, has ~1 million fixed differences to the living Asian elephant, *Elephas maximus* [45,50,63], the majority of those differences are simply neutral variation accumulated over millions of years of divergence. Only a small portion of those differences has been selected during the species’ evolution to produce the woolly mammoth’s unique adaptations. To produce a functional proxy of the woolly mammoth, the Asian elephant simply needs to be adapted to living in cold climates, which may come from only a few dozen traits derived from the interaction of only a few dozen genes and regulatory elements [63]. While the number of changes that can feasibly be made in the genome is limited, it is arguably more sensible to focus on key genes and traits than to spend resources on editing mutations that produce no effect. Using genome editing, George Church’s team at Harvard Medical School has already edited 44 loci of the Asian elephant genome and in theory the team may already have cells capable of producing suitable mammoth proxies [64].

Even when we achieve the ability to synthesize entire genomes, it may never be possible to synthesize a faithful sequence of an extinct species. The degradation of DNA over time likely precludes the ability to ever reassemble paleogenomes in their original conformity. As a result, the genomes of living species must be used as scaffolds to align ancient DNA fragments and every paleogenome can only be mapped to the synteny of the living template species. An extinct species’ exact karyotype and chromosomal synteny are biological factors entirely lost to science. Therefore, it is likely that de-extinct proxies will always be genetically different to some extent from their extinct counterparts, which lends preference to the minimalist approach that focuses on key genotype to phenotype pathways, rather than whole genome duplication.

Genetic restoration does not confer phenotypic restoration. The simplistic principle of precise hybridization is that genotype = phenotype = ecotype. But in actuality, the rule is comprehensively genotype + epigenetic conditioning = phenotype; phenotype + context = ecotype. This means that often an environmental factor or particular nurturing regime will be required to bring about the full expression of a phenotype from its genotype and acknowledging that an individual without its habitat and social structure may not exhibit its full set of traits that confer its ecotype. For example, researchers may discover a mutation responsible for hyper-sociality in passenger pigeons that fails to elicit the trait in precisely hybridized band-tailed pigeons unless they are raised in a colonial setting. Developing and employing specific conditioning protocols is not a unique process anticipated for de-extinction breeding programs but a standard and necessary component of ex situ conservation. However, since much of the context needed to replicate the phenotypes of extinct species from their genotypes was never recorded while the species was alive, proxy phenotypes may never be faithfully reproduced. Successfully achieved proxy phenotypes will be defined by de-extinction program goals as much as by available knowledge of the extinct species’ phenotypic state.

## 6. What Is a Proxy?

The classification of proxy populations is very important for navigating the regulatory pathways for introduction to wild habitats, particularly when identifying the native range to which the proxy population will be introduced [65]. A proxy species that is classified as its extinct counterpart could feasibly be regulated as a traditional reintroduction. However, environmental regulations were written for the recovery of extant species and therefore extinct species are not considered to have any present-day native range to be reintroduced, possibly presenting complications for de-extinction under present laws. If a proxy is classified as the same species as its template species and the proposed release lies outside the template species’ native range, the release would be deemed a non-native introduction, which does not inherently present regulatory barriers. But the introduction of non-native species has only been implemented in rare cases to save species whose native range is now uninhabitable. If the proxy population is considered a new species, then there is no baseline for considering appropriate range for introduction at all, which is potentially problematic as researchers are building towards a consensus that proxy populations represent new biological entities, chiefly because of the human-facilitated process from which they originate.

Some assert that the human-facilitated origins of proxy populations be reflected in their nomenclature [66]. As with the issue of epigenetics when classifying proxies, this notion can be seen as problematic when considering human influences on extant species both regarding ex situ and in situ conservation interventions. Are all captive bred species new entities? Should their nomenclature reflect that their lineage underwent a period of direct human management? Would changes in nomenclature to extant endangered species impede reintroduction efforts in the same way it is assumed that nomenclature compromises de-extinction efforts? The answers to these questions should be formulated to unite reintroduction and de-extinction under an all-inclusive regulatory framework.

What is the identity of a de-extinct proxy? If we consider de-extinction via selective breeding, the genetics of the proxy population are directly descended from a single species and therefore could be considered the equivalent of breeds or variants, rather than new entities. Domestic breeds of pigeons, for instance, exhibit such extreme phenotypic divergence that it parallels that of separate genera or families in wild species [67]; however, no one considers different breeds of pigeon to be separate species. Why should de-extinct quagga and aurochs be considered anything other than variants of extant plains zebras and cattle? This issue is less arbitrary with precise hybridization. Hybrid proxies possess genetic contributions from two separate species, making them clearly biological hybrids. Hybrids, however, have no definitive classification. For instance, non-African modern humans possess Neanderthal ancestry [46,68] but are not considered Neanderthal-human hybrids. No one has argued for splitting our nomenclature to classify human hybrids versus “pure” humans. The persistence of an extinct species genetics surviving in extant species may be more common than one may think. The recent discovery of Galapagos giant tortoise hybrids possessing ancestry of two extinct island species has formed the basis of one of the world’s active de-extinction breeding programs (Table 1) and the discovery of extinct cave bear genetics in extant brown bears now stands as the first non-human example of a Pleistocene species’ genetics surviving tens of thousands of years after its extinction [47].

A simplistic approach to the species concept is to consider how biologists typically discriminate two populations holistically. Do the two populations occupy distinct habitat zones, or niches, within the same or separate geographic ranges? Do the two populations exhibit different morphology and/or physiology? Do the two populations exhibit different behaviour? Do the two populations avoid interbreeding? Can the two populations be genetically distinguished from one another? If the answer to all or most of those questions is yes, then any biologist will consider the two populations as separate species or separate subspecies, depending on how divergent the traits happen to be. The goal of de-extinction practitioners is to produce proxy populations that, when compared to their template predecessor species, check yes to most or all of those questions. The goal is that the proxy species and its extinct counterpart share more in common than the proxy and the template. While selectively bred proxies may not exhibit genetic distinguishability from template species and hybrid proxies may only possess a handful of diagnostic single nucleotide polymorphisms (SNPs) from their extinct counterparts, it is the accumulation of behavioural, morphological and physiological phenotypes that will collectively distinguish proxies as unique entities.

With the knowledge that proxies, even hybrid proxies, are not directly descended from their extinct counterparts, it is most appropriate to consider proxy populations as new species or subspecies stemming from their extant template species with nomenclature reflecting their de-extinction process. But rather than introduce Latin and Greek roots to reflect human activity, such as *Mammuthus genosynthetica* or *Mammuthus anthrogenesis*, de-extinct proxies could simply reflect the process by which they were generated by designating selectively bred proxies as subspecies and hybrid proxies as species. Their nomenclature would be more aesthetically appropriate by calling attention to the proxy’s nature, rather than emphasizing human roles, which would once again call into question the taxonomic designations of other species whose continued existence have been facilitated by human intervention. A de-extinct aurochs could be considered the subspecies *Bos taurus feroredivivus*, the “wild aurochs reborn.” For hybrid proxies, such as the passenger pigeon, the name could be *Patagioenas neoectopistes*, the “new wandering pigeon of America,” reflecting the extant template species lineage of the band-tailed pigeon, *Patagioenas fasciata* and the extinct genetic lineage, *Ectopistes migratorius*.

## 7. Prioritizing De-Extinction Candidates

The resources required for de-extinction of any form are extensive. De-extinction programs span decades and demand large investments both financially and in interdisciplinary research and personnel. For these reasons it is important that practitioners pursue de-extinction projects in which the investment is justifiable. Much of the debate concerning de-extinction is driven by the poorly defensible argument that de-extinction diverts funding from “proven” forms of conservation—an argument that is only merited if de-extinction is considered a new conservation practice, which it is not (both points addressed later on in detail).

Academically speaking, every extinct species is a potential de-extinction candidate; however, certain technical criteria as well as ecological and social considerations (e.g., notably providing adequate welfare to the individual organisms and populations involved in the process, a consideration that overlaps all three categories of discrimination), need to be taken into account to determine whether a species is a good, poor, or implausible candidate. In 2013, Revive & Restore published a webpage of de-extinction candidate species to enable the vetting of de-extinction candidates. The list has caused a large degree of confusion because many authors assumed all of the species listed were “good candidates” for de-extinction or were being seriously considered for de-extinction programs. However, the list was paired with a criteria matrix webpage with a series of questions to assess the quality of a candidate—with the intention that viewers could readily see that some extinct species are good candidates for de-extinction while others are poor candidates and others still are impossible candidates. These pages have since been removed but have left a lingering unintentionally negative impact on the de-extinction dialogue.

There are a number of justifications for de-extinction science, from moral obligation, to scientific advancement, to stimulating social interest in conservation—the ‘wow’ factor [69,70]. However, for de-extinction practitioners the focus is heavily on ecological benefits in conservation, driving a dialogue of “species selection” that mirrors the same criteria for investing in conservation interventions for extant endangered species. A number of authors have specifically addressed candidate selection [71,72,73,74,75]. Seddon et al. [71] published the first candidate selection criteria in which the full spectrum of technical, ecological and social considerations were poised as a ten-question evaluation. Overall, the criteria are comprehensive and usefully applicable. McCauley et al. [75] present a more in depth candidate selection process focused on ecological function. These authors generally agree that if proxies of an extinct species can be feasibly generated, then the prioritization of extinct species falls to ecological significance or sociopolitical factors that will elicit a net conservation gain relative to the investment cost.

The optimal way to produce conservation gain is to focus on species that have disproportionate impacts on their ecosystems. For this reason, the top priority species for de-extinction efforts are ecosystem engineers and keystone species. Ecosystem engineers are species whose activities transform habitat structure in ways that are used by other species, supporting higher diversity than when their engineering activities are absent [76]. Typically, these are species whose biomass creates large effects, although population abundance or large body size is not a universal characteristic of engineers. For example, beavers are an iconic example of ecosystem engineers [77]; a single beaver family creates an entire wetland habitat supporting hundreds of species and impacting a large geographical area. Keystone species, in contrast, typically form a small component of an ecosystems biomass but impose trophic cascades in a community that support higher biodiversity than when they are absent [78]. Apex predators commonly assume the role of keystone species through top-down trophic forces. When reintroduced to the Greater Yellowstone Ecosystem, wolves restored key relationships with prey species, which in turn has supported high densities of scavenging species [79] and has altered vegetation dynamics in the park, allowing the return of beaver and even changing the flow of river systems [80,81]. Engineers and keystones can be distinguished as follows: a keystone species is the foundational support for an entire community network, which can influence the physical structure of the community’s habitat indirectly, while an engineer directly impacts the physical structure of a community’s habitat.

There are several other types of species that produce net gains for investment that are not necessarily ecologically significant. These are umbrella species and indicator species. The umbrella species concept has become a widely used tool for conserving biodiversity. The premise is that managers focus on saving a single species whose habitat needs overlap that of many other threatened species, casting a protective “umbrella” for which all species underneath benefit [82]. A famous and effective umbrella species is the giant panda, *Ailuropoda melanoleuca* [83]. Indicator species are quite different. An indicator species serves as a convenient monitoring tool for an ecosystems’ overall health. Indicator species are easy to locate and track and are sensitive species involved in many species interactions within the community [84,85]. The presence of a viable indicator species population serves as a surrogate measure for the overall biodiversity of an ecosystem; if the indicator species is present and thriving, it is likely that many other rarer and more elusive species are also present.

Current de-extinction programs have been criticized for focusing on “charismatic” species rather than ecologically significant species. But in reality, most de-extinction efforts are working to create notably ecologically significant proxies. However, the choice to focus on charismatic species is not a phenomenon unique to de-extinction projects. Conservation funding stems from public support, which only exists with public interest in conservation activities. Charismatic species serve to stimulate public interest. Known as flagship species, charismatic and beloved species are a mainstay of conservation campaigns. It has been argued that extinct species can serve as important flagships, not only for the potential to “bring them back” [86]. For example, the rapid decline and extinction of the passenger pigeon was the catalyst for the modern conservation movement in the United States at the turn of the 20th century [87]. None of these species-demography conservation concepts are mutually exclusive; it is possible for a single species to be a flagship, an umbrella and a keystone species. The highest priority candidates for innovating new de-extinction techniques are those that can serve multiple purposes to create conservation gains both socially and ecologically.

## 8. Active De-Extinction Breeding Initiatives

There are currently seven active de-extinction projects globally: the quagga (*Equus quagga quagga*), Aurochs (*Bos taurus primigenius*), Floreana Island giant tortoise (*Chelonoidis elephantopus*), woolly mammoth, passenger pigeon, heath hen (*Tympanuchus cupido*) and an effort to restore diverse moa species (order Dinornithiformes) (Table 1).

The quagga project is the longest running de-extinction effort, started in 1987. Quagga DNA was actually the first ancient DNA ever sequenced, the results of which indicated it is a subspecies of extant plains zebras [88], a conclusion used as a supporting tenant for initiating quagga de-extinction. Stripe-pattern variation in living plains zebras formed the foundation for an artificial selection program to reproduce individuals with similar patterning to the extinct quagga, which has recently been achieved [89]. Dubbed “Rau quagga,” several individuals have been released to roam freely on the Nuwejaaars River Nature Reserve in South Africa. Rau quaggas possess the same stripe patterning as their extinct counterparts but so far lack the same coloration. While the goal is to reintroduce quagga proxies to their former habitat, which will restore biodiversity and the grazing ecology of equids in the region, this goal could arguably have been accomplished by simply introducing unaltered, non-migratory zebras to the Karoo basin. It is difficult to defend the position that changing a zebra’s stripes influences its ecological role in the environment but nonetheless quagga de-extinction increases zebra phenotypic diversity.

Aurochs de-extinction programs are using back-breeding to reconstitute the phenotype of the Aurochs, the ancestor of all living cattle breeds. Several initiatives to back-breed Aurochs began recently as part of the larger Rewilding Europe Project; however, the earliest attempts to back-breed Aurochs date to the 1940s, predating the quagga project by over four decades. The idea of back-breeding aurochs goes back further still to the early 1800s, making it possibly the first conceived program to use breeding to accomplish the restoration of an extinct species [90]. The goals of the project are explicitly to restore the ecological role of the species, whose historic grazing was instrumental to maintaining grassland patch dynamics throughout Eurasia. The project certainly fits the general criteria of de-extinction, replacing an extinct population with an extant population; however, from a genetic perspective the Aurochs is not actually extinct, it is simply domesticated [53]. Domestic cattle breeds all possess various ancestral and derived mutations and phenotypes due to selective breeding but as a lineage these are all direct descendants of the wild Aurochs, meaning the lineage, however phenotypically modified, is unbroken (Figure 1). Although, because of artificial selection, unless the derived mutations of modern cattle breeds are edited out of the proxy population it will still represent a slightly different genetic composition to the ancestral Aurochs. The Aurochs is an excellent example of the types of questions, stated earlier in this paper, that de-extinction creates: was the Aurochs bred into extinction (extinction through evolution), or are living cattle breeds actually Aurochs? Had wild Aurochs populations not gone extinct in the 17th century, they would undoubtedly have undergone selection for particular alleles in adapting to increasingly human-impacted habitats as well as accrued new mutations with new generations. Paleogenomics reveals that historic wild Aurochs possessed a large degree of genetic variation, which would have been divided into a metapopulation structure with restricted gene flow due to modern human imposed barriers, producing genetically distinct subpopulations. Such subpopulations would arguably require some degree of human management to conserve, as is the case with many species living in highly developed environments. In many respects, the many genetic variants of domestic cattle today reflect the genetic demography of what could have been expected in wild populations had they survived to modern times. Ultimately, the original collective aurochs’ phenotype in conjunction with its ecotype no longer exists and it is an excellent case of anagenetic extinction. Though the genetic lineage does not need to be recovered, as it has never been lost, recovering the original phenotype and ecotype requires programmed breeding, squarely classifying aurochs restoration as de-extinction rather than recovery. Sadly, it appears that aurochs breeding programs are waning, with interest declining in the past two years. Breeding herds have been disbanded and Rewilding Europe, which had a contract to use Tauros cattle, has ceased using Tauros cattle in all areas in which they work [91]. It is unclear at this time if Aurochs de-extinction programs are still active and intend to fulfil their original goals.

The aurochs and quagga programs represent some of the only de-extinction programs in which traditional breeding methods can be feasibly applied. However, as exemplified by the three-decades run of the quagga program, traditional selective breeding is a long and inefficient process, yielding only a few select high value individuals each generation among many offspring displaying off-target phenotypes. Since both the quagga and aurochs genomes have been sequenced (Table 3) and reproductive technologies are highly optimized for bovine and equids, precise hybridization would yield far more effective results. Given that the quagga and aurochs are extremely genetically close to their living relatives/descendants, precise hybridization from the start of gene-editing in a cell line to the birth of the desired phenotypic proxy, for either aurochs or quagga, could be performed in less than five years with ample effort. Precise hybridization in such a short time frame would also be far less expensive than decades of animal husbandry costs. For the quagga specifically, precise hybridization could produce the quagga’s unique coloration, which selective breeding to date has not yet replicated.

It was recently discovered that hybrid descendants of the extinct Floreana giant tortoise survive both in captivity and in the wild [92]. These hybrids are a mix of Floreana giant tortoise, which have saddle-backed-shaped shells and tortoise species with dome-shaped shells. A number of hybrids exhibit the saddle-backed phenotype, which allows them to browse a wider range of vegetation than domed species. A number of hybrids were captured from the wild and transferred to captivity to initiate a breeding program that intends to cross-breed hybrids in a manner to reduce non-Floreana tortoise ancestry, thereby restoring the Floreana tortoise genome. Miller et al. [92] state that this is a form of de-extinction but also argue that the Floreana giant tortoise is not extinct, owing to significant genetic representation of the species in the hybrids, many of which could be first generation hybrids given the longevity of giant tortoises. From a genetic perspective, it is likely that the Floreana giant tortoise genome, which evolved in isolation on Floreana island and adapted to the local environment, does exist in its entirety, simply parsed out in different portions among hybrids. For comparison, Neanderthal representation in modern humans is minute, accounting for only ~1% [68]. This presents a conundrum for the definitions of de-extinction and recovery outlined here; while the recovery of the Floreana giant tortoise will be facilitated by a manipulated breeding pedigree, the majority of the Floreana giant tortoise genome, if not all of it, survives. Therefore, this may actually be species recovery aided by de-extinction techniques, rather than actual de-extinction. It is hard to argue that tortoises that exhibit Floreana phenotypes, possess a majority of Floreana genetics and are the actual descendants of Floreana giant tortoises, can be considered proxies rather than the original species. As stated earlier, minor amounts of hybridization are not sufficient grounds to reclassify species, so tortoises that are mostly Floreana would arguably be classified as *C. elephantoidis*. If the goal is to save the Floreana giant tortoises’ unique evolutionary lineage, this is an excellent project worth the investment of decades; tortoises take 20–25 years to sexually mature in captivity, meaning the program could encompass centuries of breeding. However, from an ecological goal-oriented viewpoint (the goal of de-extinction) it would be immensely cheaper and faster to simply transfer saddle-backed tortoises, whether they are hybrids or not, to Floreana island, to resume the extinct species role in the environment. As noted earlier, Aldabran tortoises were introduced to replace extinct Mauritian giant tortoises to great ecological success [93].

All other current de-extinction projects are employing precise hybridization to accomplish their goals. What separates the ongoing precise hybridization projects from other de-extinction projects is that none of the species of current precise hybridization programs can be plausibly replaced with living species. Elephants cannot survive in cold environments as mammoths once could, no living ratite species possesses the same beak or feeding range of various moas and no living pigeon species breeds colonially, which is the key trait that made the passenger pigeons ecologically important. The heath hen and its living relative, the greater prairie chicken, are visibly indistinguishable but greater prairie chickens were released to the heath hen’s former range in the late 1800s to early 1900s to no success. Conservationists now know that greater prairie chickens cannot survive in isolated populations on small patches of suitable habitat, the very conditions in which extinct heath hen metapopulations persisted. When a living species cannot replace the ecological function of an extinct species, programs must resort to using precise hybridization, which was not a feasible technique until the advent of efficient and cost effective genome editing via CRISPR-Cas9 [94]. Restoring proxies of these extinct species requires a mixture of alleles from the original extinct species and careful conditioning during breeding programs to elicit the right phenotypes to successfully introduce ecotypes of elephants, emu, band-tailed pigeons and prairie chickens capable of resuming their extinct counterpart’s ecological functions.

Aurochs, Floreana giant tortoise, woolly mammoth and passenger pigeon were all ecosystem engineers. Each species’ grazing, browsing and destructive activities shaped their habitats in ways that benefited other wildlife from plant community composition to availability of resources. Giant tortoises act as the primary megaherbivores of every island on which they live throughout the Indo-Pacific region, influencing the faunal compositions of entire ecosystems, via grazing, seed dispersal and nutrient transport [95]. As New Zealand’s megafauna, moa likely had similar major impacts on plant community composition, dispersal and nutrient transport, though researchers do not find significant evidence that the species’ impacts engineered habitat structure [96,97]. Aurochs and Mammoth grazing stimulated the competitive advantage of grasses over other plants, keeping grasslands maintained and productive. In the absence of grazing megafauna, the former Pleistocene mammoth steppes of Eurasia and North America have been overtaken by Holocene tundra and taiga [98]. Habitat conversion experiments by Sergey Zimov in Siberia have shown that the return of grazers stimulates the conversion (engineering) of tundra to grassland [99]. Among the grazing species that can recolonize and convert tundra to grasslands (deer, bovine, antelope, horses) none can assume the supermegafaunal role of elephantids, which have different grazing/browsing impacts, different nutrient transport effects and are the only animals large enough to open up taiga forests for grassland conversion by toppling trees. Dense flocks of passenger pigeons created forest disturbances, by clearing undergrowth (guano deposition) and creating canopy gaps (overcrowding branches), perpetuating forest regeneration cycles and maintaining the presence of successional habitats for tens of thousands of years before their demise in the 1880s [100]. Today, successional habitats are rare and declining throughout eastern North America, leading to declines in dozens of plant and animal species [101,102,103,104,105,106]. Human interventions designed to produce successional habitats are costly and infeasible to scale up to meet conservation needs [101,107,108,109]. Restoring passenger pigeons offers a more effective solution.

The activities of passenger pigeons, aurochs and mammoths also stimulated higher rates of photosynthesis, which created carbon sinks in their environments. Carbon storage to combat climate change is the primary driver for woolly mammoth de-extinction. Not only do grasses sequester and store carbon more effectively than forests and most other ecosystems [110], the activities of grazers on tundra grasslands allow cold winter air to reach the ground surface and keep permafrost frozen. During the summer the extensive root system and high albedo of grasses keep the permafrost insulated. Melting permafrost represents the largest source of hydrocarbon release on earth. Habitat conversion through grazing is not a fast enough solution to prevent the exacerbation of climate change from permafrost melt, which will require technological solutions and major infrastructure changes to human societies to solve but the conversion of tundra to grasslands offers a long-term buffer to severe climate fluctuations, a service the mammoth steppe provided for hundreds of thousands of years through major climate cycles before its disappearance at the onset of the Holocene. The viability of the mammoth steppe providing a climate fluctuation buffer is, however, wholly dependent on scale. Proponents of tundra-to-grassland conversion, including mammoth de-extinction practitioners, recognize and support the conversion of immense tracks of tundra throughout Eurasia and North America.

**Figure 2:**
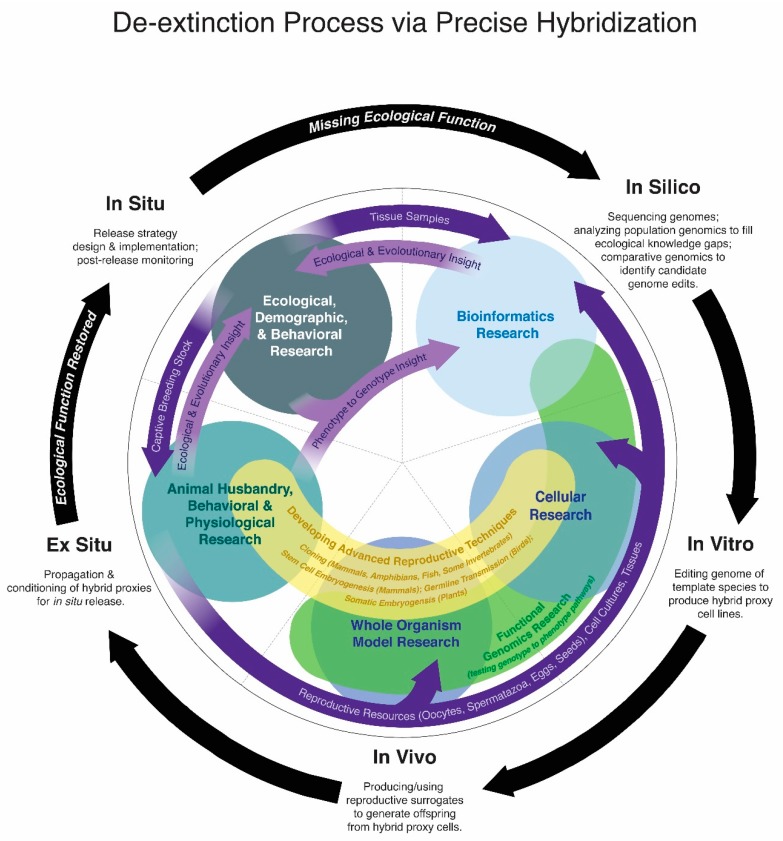
The de-extinction process via precise hybridization. The sequential stages begin with in silico and end in situ, shown on the outside circle. The inner circle shows the compartmentalized and overlapping supporting research for the de-extinction process, with arrows showing exchange of resources (dark purple are physical resources, light purple are knowledge resources). Passenger pigeon de-extinction is a model example of this research process, the genomes of the template species were sequenced from a wild individual (in situ), a captive individual (ex situ) and embryonic fibroblast cultures (in vitro), [58]. The sequencing of those genomes has been key to understanding the species’ role as an ecosystem engineer [100], (in silico to in situ insight pathway). Currently, program collaborators are using newly developed bioinformatic pathways [111] to identify candidate passenger pigeon alleles for de-extinction purposes using a comparative genomics process that compares species pairs based upon shared/derived phenotypes (in situ to in silico insight pathway). The species’ ecology has been clarified further through ex situ observations of band-tailed pigeon digestion and in situ experiments to understand seed dispersal and predation [112]. The program has studied the animal husbandry of the template species [113] and used tissues from one of the Bronx Zoo individuals of that study to improve the Murray et al. genome sequence to a chromosomal level assembly (unpublished). Recently the program has begun genetic engineering research using domestic pigeons to facilitate functional genomics research in vivo [114]. Domestic pigeon primordial germ-cell cultures have been preliminarily tested for advancing the eventual in vitro to in vivo progression to create hybrid proxies; the pilot study did not successfully culture cells long term but produced data for staging embryonic development for optimal isolation of germ cells [115].

De-extinction via precise hybridization includes five stages of work (Figure 2), and while these stages do form linear milestones, there is overlapping foundational research for each stage. For instance, animal husbandry research is necessary to use living template species both for in vivo and ex situ stages and for birds the in vitro stages require acquiring resources from breeding stocks of the template species. Understanding the ecology of an extinct species is greatly informed by the population dynamics that can be modelled from genomic data and discovering genotype to phenotype relationships requires functional genomic testing (in vitro and in vivo). Identifying candidate alleles for genotype to phenotype discovery is a process that can be greatly assisted by knowledge of the template and extinct species phenotypes, which can only be discerned in many cases from in vivo, ex situ and in situ research, including historical observations, paleobiological and paleoecological modelling for extinct species.

Each precise hybridization program is currently at different stages. The moa project’s long-term goal is to sequence genomes of all nine species (in silico) to comprehensively research moa paleoecology, primarily to inform conservation decisions for whole ecological communities on New Zealand and also to eventually guide de-extinction of moa species (in silico to in situ insight) [116]. To date only a single moa species’ genome has been sequenced: the little bush moa, *Anamalopteryx didiformis* [52], sequenced independently of de-extinction efforts, nonetheless this genome stands as the first concrete resource on the path towards moa de-extinction as the project works to sequence additional species. In contrast, multiple individual genomes of the heath hen, passenger pigeon and woolly mammoth have been sequenced to date [45,58,59]. Comparative genomics to identify candidate alleles for genotype to phenotype research is ongoing for all programs. The heath hen program has initiated in vitro/in vivo research to establish primordial germ cell cultures for germ-line transmission, a necessary step for reproducing gene-edited birds. The woolly mammoth program has already edited 44 loci in vitro and is currently researching stem cell embryogenesis pathways in mice for the in vivo phases of de-extinction [64,117]. The passenger pigeon program has established foundational animal husbandry research for the in vitro, in vivo and ex situ stages [113,118] and is currently working to develop a strain of domestic pigeons for model functional genomics research [119]. Significant ecological (in situ) insight for passenger pigeon de-extinction has been gained from in silico genomic data [58] and ex situ/in situ dietary experiments [112].

## 9. Criticisms of De-Extinction via Breeding

Dozens of criticisms have been highlighted in recent de-extinction literature; here I will describe how a comprehensive understanding of de-extinction practice refutes the four most common criticisms of de-extinction argued in both in peer-reviewed and populist literature. I do not consider animal welfare concerns in this manuscript, as these concerns are not inherently unique to de-extinction via breeding. Animal welfare and de-extinction via breeding concerns are shared with all ex situ and in situ conservation programs but include also the animal welfare concerns of the use of animals in laboratory research, making the topic too complex to be treated briefly and is the focus of a book chapter currently in preparation [120].

### 9.1. De-Extinction Is “New”

De-extinction via breeding has been widely presented by many authors as a novel conservation endeavour wholly separate from historic conservation trends, despite the fact that its relationship to reintroduction and replacement efforts were published early on [25,26,121]. When considering that translocation of extant species to replace extinct populations is a form of de-extinction (dating back to the 1830s), then de-extinction is a conservation intervention that predates many practices typically considered as “traditional” conservation, that is, habitat protection, harvest bans and regulated game management, all of which were first implemented for conservation purposes between the late 19th and early 20th centuries. Dozens of translocations to replace extinct populations have been performed globally [26]. Aside from the well-known case of wolves in Yellowstone National Park, reintroductions via translocation in the United States include: beaver, bighorn sheep in badlands habitats, elk in eastern states and bald eagles and wild turkey in New England. Wild turkeys, which are abundantly common now in New England, had gone extinct in the region by the 1840s and were absent until successful reintroductions in the 1970s, ~130 years later [122,123].

The idea that de-extinction is new is rooted in the belief that the breeding techniques being employed are novel. The processes of artificial selection and genetic engineering may be new to conservation but both breeding techniques are established processes. Artificial selection has been done for millennia, producing most of the foundational research for modern genetics and evolution. While precise gene editing is a new innovation [94,124,125], genetic engineering dates to 1974 [126]. Genetically engineered organisms are now used to produce medical products (e.g., insulin), biofuels and bioplastics. Genetically engineered organisms are used every day in scientific research that advances medicine and agriculture and genetically engineered food products have been consumed by billions of people for decades. While it is true that the effects of genetic engineering and gene editing of most genes and genetic regions await discovery, the tools and process of discovering genotype to phenotype relationships are not radical and unpredictable technologies, they are well-characterized and ever-improving tools.

Overall, criticizing de-extinction because it is experimental is grossly myopic; all forms of conservation intervention are experimental. No model simulation can ever account for every variable within an ecosystem, meaning in situ results of intervention are always subject to unknown outcomes. The experimental nature of conservation has not been an insurmountable obstacle to reintroductions and ecological replacements in the past but instead has shaped an ever improving scientific discipline [28]. No de-extinction practitioner has advocated the in situ release of ex situ-bred proxy populations without undergoing the same scientific and regulatory processes that all reintroduction programs undergo before and after release. This fact is being more widely appreciated by those debating de-extinction and debates have shifted notably from the “should we, should not we” orientation to “when should we and how should we” proceed with de-extinction.

### 9.2. Extant Species Should Be Given Priority

Many authors support research into de-extinction biotechnologies for the purpose of aiding extant endangered species, which they argue should be given conservation priority. This is a sound argument, given that so many extant species are threatened; however, two additional points must be considered:

Eliminating conservation threats for many species requires the restoration of ecological processes or species-interactions left vacant due to extinctions. For example, seed dispersal and pollination of many island plant species have declined or virtually ceased owing to the extinction of endemic co-evolved mutualists, resulting in declines and major population demographic shifts [127,128,129,130,131]. These plants cannot be conserved without restoring seed dispersal and pollination, for which some interactions are so co-evolved with specific endemic extinct fauna that de-extinction of a suitable proxy may be the only pathway to in situ recovery independent of human facilitation.

The experimental process of developing reproductive techniques for de-extinction demands excess resources to optimize, which rare endangered species cannot provide. Avian reproductive technologies are an exemplary case study of the value of de-extinction as a model. The only viable advanced reproductive technique for birds is germ-line transmission of cultured primordial germ cells (PGCs) [132,133]. Developing optimum conditions to culture PGCs requires experimental sets of embryos for isolating PGCs and testing varying culture media. Revive & Restore’s heath hen de-extinction program recently succeeded in culturing late-stage germ cells (non-primordial), an initial milestone resulting in five cultured cell lines from an experimental set of thirty-six fertile greater prairie chicken eggs (data unpublished). Obtaining primordial stage germ cells will require further experimental sets of eggs. PGC technologies are the means to genetic preservation, maintenance and recovery for avian species but one would not consider diverting thirty-six fertile eggs for PGC culture experiments from the endangered kākāpō parrot (population currently 148 individuals, per New Zealand Department of Conservation [134]). When considering the laboratory research required to advance biotechnologies for genetic rescue, extinct species are ideal model systems for one main reason: they are extinct. When experimental trials fail, a species’ fate is not on the line. Almost all template species for active de-extinction breeding programs are non-threatened species (Table 1) and in cases in which the template species is endangered, de-extinction scientists are pursuing reproductive technologies that will eliminate the need to use surrogates from the template species [120]. De-extinction offers a compelling reason to work with wildlife species that otherwise are not of conservation concern or scientific significance as model organisms, developing transferable genetic rescue applications without conservation loss.

### 9.3. Recently Extinct Species Are Better De-Extinction Candidates

Several authors argue that de-extinction should only be applied to recently extinct species. This is an assertion that can be undercut with one example: extinction due to habitat loss. For species that have gone extinct due to habitat destruction or alteration, there is no justifiable reason to pursue de-extinction until the habitat has been restored. In the case of ecosystem engineers, habitat restoration will be directly facilitated by the restoration of de-extinct proxy populations, but the habitat still needs to be in a state suitable for the introduction of the proxy population. Habitat loss is an ongoing issue for many declining and recently extinct species, whereas species that went extinct earlier in history, particularly between the 16th and 19th centuries, were extinguished due to human harvest, a practice now regulated in most terrestrial environments. For example, the extinction threat of the passenger pigeon—industrial scale harvest—has been eliminated and the habitat is suitable for introduction after decades of reforestation. In contrast, the threat of mosquito-borne disease precludes de-extinction of dozens of extinct Hawaiian birds, regardless date in which they went extinct: the passenger pigeon went extinct in 1914, whereas the Hawai’i ‘Ō‘ū honeycreeper, Kaua’i ‘Ō‘ō honeyeater and Kāma‘o thrush all went extinct in the 1990s [135] and even more recently the Po’ouli honeycreeper went extinct in 2004 [136].

This notion that recently extinct species are more appropriate to restore also ignores successful reintroduction and rewilding efforts around the globe. Beaver were absent from Great Britain for ~400 years when reintroduction began, which ultimately resulted in overwhelming positive benefits [137]. The grazing mammal ecotypes brought together to create an ecological community at Oostvardersplassen, in the Netherlands, have not existed in the region for over 1400 years [138]. Some of the ecotypes converting tundra to grassland at Pleistocene Park have not inhabited the region in 2000 to 14,000 years [139]. California condors were successfully released in Grand Canyon National Park to save the species, an area in which condors have not lived in over 10,000 years [140].

### 9.4. De-Extinction Detracts Funds from “Proven” Conservation and Endangered Species

The weightiest criticism is that de-extinction detracts funds from other conservation efforts. Bennett et al. [141] published the first model simulation of the impacts of de-extinction spending on conservation, using New Zealand and Queensland, Australia as hypothetical case studies. Popular press toted the publication as evidence that de-extinction will cause more extinctions than it reverses/prevents [142,143,144]. However, this conclusion was found only in one of the three model simulations, in which government conservation funds available to extant species were diverted to de-extinction, a fictional scenario. In model simulations when some or all funds for de-extinction are independent of conservation resources, conservation as a whole gained rather than lost. In building constructive de-extinction debates, several points are important to note. The first being that all funds explicitly used for de-extinction efforts have been donated from private individuals supporting non-profit organizations; the majority of donors to Revive & Restore’s de-extinction efforts are investors in technology sectors [145], representing new money for conservation purposes. Here it is worth emphasizing that this is not new money for de-extinction but that almost all founding funders to Revive & Restore have contributed donations towards the broader mission of the organization: the development of biotechnologies for genetic rescue. These donors are fuelling research for important innovations from which de-extinction as a conservation tool builds but de-extinction is not the sole conservation goal of these innovations. Revive & Restore’s de-extinction programs are intentionally designed to advance biotechnology for broader conservation applications. A second discrepancy with Bennett’s studies and other critics’ assertions, is that de-extinction is treated as a separate competitive discipline at odds with conservation, rather than the conservation tool that it is. A third, most important factor, when considering Bennett’s results, is the context. As island groups, both Australia and New Zealand have suffered extensive extinctions and an influx of hundreds of invasive species, resulting in grossly altered habitats and communities. Particularly in New Zealand, many endemic species presently survive only within predator-exclusive enclosures and on predator-free satellite islands. Many of these species survive outside of their historic native range, to which they cannot feasibly return in the near future. It is hard to imagine that proxies of specialized extinct endemics would have chances of recovery under such conditions. The extant endangered species of New Zealand represent an abnormally expensive group of species to conserve compared to other global conservation regions. Lastly, Bennett et al. did not apply any of the previously published candidate selection criteria to the extinct species used in their models, even though the selection criteria in Seddon et al. 2014’s publication shares co-authors with Bennett’s study. None of the species used in Bennett’s study would be considered viable candidates in light of those prioritization criteria, rendering Bennett’s study entirely academic rather than practically informative.

Yet another issue with drawing conclusions from Bennett et al. [141] is that the study only considered conservation cost/benefit during the ex situ and in situ stages of de-extinction. Ex situ conservation is inherently expensive regardless of the taxa being maintained for in situ recovery. Many ex situ programs in the United States have cost billions of dollars each over several decades of operation [146]. When considering de-extinction, conservationists must also account for the cost-to-benefit value of the in silico, in vitro and in vivo stages. A great deal of the initial expenses of de-extinction, particularly in silico research, is completely independent of conservation science. The genomes of extinct species are sequenced regardless of their use by de-extinction programs, as evidenced by the many genomes now published and publicly announced at conferences (Table 3). The discoveries from ancient genomes have yielded many valuable discoveries that shape our understanding of evolution and ecology and provide insights to functional genomics. The only genome of an extinct species sequenced to date exclusively as part of a de-extinction effort is the heath hen genome [59], which will soon be joined by the various moa genomes as additional de-extinction motivated sequencing efforts. However, heath hen de-extinction is being managed as a comprehensive genetic rescue genomics program for all *Tympanuchus* species, producing the first genomic resources for the threatened lesser prairie chicken, *T. pallidicinctus* and the critically endangered Attwater’s prairie chicken, *T. pinnatus attwaterii*. While not endangered, isolated populations of the greater prairie chicken, *T. pinnatus pinnatus*, require perpetual genetic diversity augmentation via translocation to prevent extinction vortexes from inbreeding [147,148,149,150]. The advanced reproductive technologies in development with greater prairie chickens will allow the genetic preservation of unique populations as well as revolutionize genetic rescue for the genus. In general, the reproductive technologies that are being developed for de-extinction, which leverage domestic surrogates, may reduce ex situ costs for endangered species long-term [120].

Similarly, other de-extinction programs are already producing peripheral conservation gains to extant template species. Passenger pigeon de-extinction has established the largest captive flock of band-tailed pigeons at an AZA member institution, resulting in the first published animal husbandry protocols for the species, an invaluable resource for potential ex situ intervention should it ever be necessary. The woolly mammoth de-extinction team has begun building foundational research to culture elephant herpes virus [151], a pathogen lethal to infant Asian elephants threatening wild and captive populations [152]. The pathogen has never been successfully cultured, making vaccine production impossible [153]. This biotechnology-based research to aid elephant conservation only gained momentum and funding thanks to interest in woolly mammoth de-extinction and resource overlap. Quantifying conservation gains and losses due to de-extinction is a much more complex formula than simply considering competition for ex situ and in situ funding presented by Bennett et al. [141].

## 10. Coming to a Consensus: Restoring Centricity to Constructive Dialogue

De-extinction dialogue represents a diversity of voices: from ethical philosophers, environmental lawyers and historians to population geneticists and biotechnologists. Peer-reviewed literature is almost evenly split between papers whose lead author is a social scientist or philosopher [20,21,22,25,65,69,70,73,74,154,155,156,157,158,159,160,161,162,163,164,165,166,167,168,169,170,171,172,173,174,175,176,177,178,179] and those whose lead author is a biological or environmental scientist [23,26,53,66,71,72,75,141,180,181,182,183,184,185,186,187,188,189,190,191,192,193,194,195,196,197]. Authors of every discipline have contributed to the emerging understanding of de-extinction, often times outside of their primary discipline. Papers by social scientists have presented thorough reviews of conservation biology and others authored by biologists have been entirely philosophical. If scrutinized using the IUCN definition as a baseline, conflicting and varied descriptions of de-extinction process and/or purpose emerge as the dominant issue in the literature. Partially divergent and in some cases extreme departures from the IUCN definition of de-extinction have been used by many authors to build de-extinction debates. More concerning, it appears that some authors have intentionally overlooked other publications to build their own definitions of de-extinction solely for the purpose of supporting their viewpoints. This is not a problem inherent to those that oppose de-extinction practice. Even several authors that support de-extinction research have flawed interpretations or independently fabricated definitions of de-extinction [20,154,175,198]. It is worth noting that divergent de-extinction definitions are more often, understandably, found in publications authored by social scientists and philosophers, typically trained to consider more abstract and varying components of a subject than biologists. Of publications authored by social scientists and philosophers, over half outline de-extinction stances widely differing from the IUCN definition [21,22,73,154,155,156,163,167,168,169,170,171,172,174,176,198,199], as opposed to less than a quarter of biologist lead papers [53,180,192,193,194,195]. If one re-evaluates all of the literature in light of the revised de-extinction definition provided here, then most other publications present definitions of de-extinction largely incongruent with actual practice [23,66,69,70,71,72,74,157,158,160,161,166,179,181,182,187,188,189,190], though authors’ departures to more abstract de-extinction concepts are building important philosophical ramifications for conservation science and the relationships of humans and non-human species. As conservation is a socio-political process performed by people, not strictly a biological scientific practice, delving into de-extinction from a multitude of perspectives is an invaluably constructive exercise, though it loses applicable value if authors are attempting to build a singular debate around multiple definitions.

Confusion over de-extinction practice stems largely from the absence of one key voice: de-extinction practitioners. Not one de-extinction paper preceding this manuscript has been authored by a scientist working on the de-extinction breeding programs at the centre of critical attention. The lack of peer-reviewed publications authored by de-extinction program leaders is in part because there are only seven active de-extinction programs globally, most at nascent stages of progress. Another factor is the nature of de-extinction programs, which take years if not decades to reach noteworthy milestones. In short, papers based on data will be few and far between for any de-extinction program. It is rather easy to build a body of literature based on postulation, speculation and opinion but rather difficult to build a reliable body of knowledge based on thorough reviews, model simulations and empirical data, all of which take considerable time, especially for programs that are expected to encompass entire careers or longer. Of the literature published to date, less than 10% is based upon any modelled or collected data [53,141,184,185,200] and only half root their arguments and discussions in relevant empirical or substantiated established intellectual content [19,23,25,53,65,69,71,72,74,141,155,157,159,160,161,180,182,183,185,186,187,188,189,190,191,192,201,202]. The remaining publications present many debates and perspectives within a “hyperbolic echo chamber” of de-extinction concerns without drawing from examples of reintroduction biology, animal welfare ethics, or other pertinent established fields of literature and science [20,21,22,69,73,154,158,162,163,164,165,166,167,168,169,170,171,172,174,175,176,193,194,195,196,197,198], arguably inflating problematic, possibly irrelevant, or worse, unfounded points alongside important and very real critiques.

Aside from the absence of de-extinction practitioners in peer-reviewed literature and the myriad of conflicting de-extinction definitions, a perhaps even more problematic trend in de-extinction literature is that almost all authors have often cited populist sources, building an academic body of literature founded on press articles rather than standardized, more reliable sources of information as peer reviewed literature. This comes in part from an attempt to represent the voice of de-extinction practitioners, who are commonly interviewed for press articles. But a better approach would be to interview de-extinction practitioners directly, or work collaboratively with de-extinction practitioners as co-authors, rather than relying on relayed information from select quotes or potentially agenda-tainted views of journalists.

In summation, there is a serious problem in de-extinction dialogue, which results from a lack of a central understanding from which to build. It is my hope that the definition of de-extinction practice and new innovations, as presented by a de-extinction practitioner for the first time in peer-reviewed publication, may restore centricity and focus to debates currently revolving about disparate themes. De-extinction must be considered through accurate understanding of its techniques and in the framework of its intended conservation purposes as a conservation intervention. Similarly, the debates and criticisms of de-extinction should always be viewed in relation to how they apply to conservation of extant species and ecosystems, before being presented. While recently initiated de-extinction projects and tools are new and exciting, their core themes are familiar, tried-and-true practices that have reliably produced conservation gains time and time again. De-extinction via new breeding techniques only stands to become a more efficient, expansive and versatile conservation intervention.

## Figures and Tables

**Table 1:** List of active de-extinction breeding programs.

Species	Common Name	Extinction Date	De-Extinction Technique	Conservation Category	Template Species	Template Species Conservation Status (IUCN/U.S. ESA)	Organization
*Equus quagga quagga*	Quagga Zebra	1883 AD	Artificial Selection	Biodiversity	*Equus quagga burchelli*	Near Threatened/NA	The Quagga Project
*Bos taurus primigenius*	Aurochs	1627 AD	Backbreeding of Domestic Descendants	Ecosystem engineer (grassland maintenance)	*Bos taurus taurus*	Not Assessed/NA	Operation Taurus; Taurus Project
*Chelonoidis elephantopus*	Floreana Island Giant Tortoise	Circa 1850 AD	Hybrid-backbreeding	Ecosystem engineer (megaherbivore)	*C. elephantopus* × *Chelinoidis becki*; *C. elephantopus* × *Chelinoidis hoodensis*	becki = vulnerable, hoodensis = critically endangered; U.S. ESA NA to both	Galápagos National Park Service
*Ectopistes migratorius*	Passenger Pigeon	1914 AD	Precise Hybridization	Ecosystem engineer (forest disturbance/regeneration cycle)	*Patagioenas fasciata*	Least Concern/Not Listed	Revive & Restore
*Mammuthus primigenius*	Woolly Mammoth	~3900 yr BP	Precise Hybridization	Ecosystem engineer (megaherbivore, grassland maintenance)	*Elephas maximus*	Endangered/Endangered	Revive & Restore
*Tympanuchus cupido*	Heath Hen	1932 AD	Precise Hybridization	Indicator species	*Tympanuchus pinnatus*	Vulnerable/NA	Revive & Restore
Anamalopteryx didiformis *	Little Bush Moa	15th Century AD	Precise Hybridization	Megaherbivore	*Dromaius novaehollandiae*	Least Concern/NA	Genetic Rescue Foundation

* The Genetic Rescue Foundation intends to restore proxies of all nine extinct moa species. The Little Bush Moa is simply the first species with a sequenced genome and serves as a stand in here. AD: anno domini; BP: before present; NA: not applicable.

**Table 2:** Evolutionarily torpid species/subspecies.

Species	Common Name	Multi-Celled Individuals	Cryopreserved Cultured Cells and/or Tissues	Active Recovery Program	Reference
*Ceratotherium cottoni*	Northern White Rhinoceros	2	Yes	Yes	[34,35]
*Capra pyrenaica pyrenaica*	Bucardo	0	Yes	No	[24]
*Lipotes vexillifer*	Yangtze River Dolphin	0	Yes	No	[38]
*Melamprosops phaeosoma*	Po’ouli Honeycreeper	>2	Yes	No	[39]
*Chelonoidis abingdoni*	Pinta Island Giant Tortoise	0 *	Yes	No	[39]
*Rheobatrachus silus*	Gastric Brooding Frog	0	Yes	Yes	[37]
*Ecnomiohyla rabborum*	Rabb’s Fringe-limbed Tree Frog	0	Yes	No	[39]
*Achitinella apexfulva*	Oahu Tree Snail Sp.	1	Yes	No	[39]
*Partula faba*	Captain Cook’s Bean Snail	0	Yes	No	[40]
*Partula turgida*	Raiatean Thin-shelled Polynesian Snail	0	Yes	No	[41]

* There are extant hybrid giant tortoises with *C. abingdoni* ancestry.

**Table 3:** List of palegenomes of extinct species/subspecies.

Species	Common Name	Extinction	Reference
*Homo sp.* ^‡^	Sima de los Huesos hominins	Pleistocene	[43]
*Homo sp. Altai*	Denisovan Man	Pleistocene	[44]
*Palaeoloxodon antiquus*	Straight-tusked Elephant	~35,000 yr BP	[45]
*Homo neanderthalensis*	Neanderthal	~28,000 yr BP	[46]
*Ursus spelaeus* *	Cave Bear	~24,000 yr BP	[47]
*Camelops hesternus* ^‡^	Camelops	~13,000 yr BP	[48]
*Haringtonhipppus francisci* ^‡^	New World Stilt Legged Horse	~12,000 yr BP	[49]
*Mammuthus columbi* *	Columbian Mammoth	~10,900 yr BP	[45]
*Mammut americanum* *	Mastodon	~10,000 yr BP	[45]
*Mammuthus primigenius*	Woolly Mammoth	~3900 yr BP	[50]
*Camelus dromedaries* ^‡^	Wild Dromedary Camel *	~2000 yr BP	[51]
*Anomalopteryx didiformis* *	Little Bush Moa	15th Century AD	[52]
*Bos taurus primigenius*	Aurochs	1627 AD **	[53]
*Raphus cucullatus* *	Dodo	Late 17th Century AD	[54]
*Pinguinus impennis*	Great Auk	1844 AD	[55]
*Equus quagga quagga*	Quagga Zebra	1883 AD	[56]
*Ectopistes migratorius*	Passenger Pigeon	1914 AD	[57,58]
*Tympanuchus cupido* *	Heath Hen	1932 AD	[59]
*Thylacinus cynocephalus*	Thylacine	1936 AD	[60]

^‡^ Only small portions of the nuclear genome have been sequenced. * Genomes sequenced to low coverage (≤5x. ** While widely accepted as the extinction date of the nominate aurochs, is has been argued, as outlined in Section 8, that the aurochs is not extinct as its descendent domestic subspecies *B. t. taurus* and *B. t. indicus* survive, reflected by the domestication event in Figure 1.
